# Single-Stage Posterior Vomerine Ostectomy, Premaxillary Setback, Bilateral Gingivoperiosteoplasties and Primary Bilateral Cheiloplasty in Patients with Protuberant Premaxilla

**DOI:** 10.3390/jcm13092609

**Published:** 2024-04-29

**Authors:** Usama S. Hamdan, Jose A. Garcia Garcia, Mario S. Haddad, Robert A. Younan, Antonio M. Melhem, Rami S. Kantar, Wassim W. Najjar

**Affiliations:** 1 Global Smile Foundation, 106 Access Rd, Ste 209, Norwood, MA 02062, USA; jgarcia@gsmile.org (J.A.G.G.); mario.haddad7@gmail.com (M.S.H.); robert.a.younan@gmail.com (R.A.Y.); antonio.melhem95@gmail.com (A.M.M.); ramikantar@gmail.com (R.S.K.); wnajjar@gsmile.org (W.W.N.); 2 Department of General Surgery, Hackensack Meridian Health Palisades Medical Center, North Bergen, NJ 07047, USA; 3 Department of General Surgery, Medical College of Georgia, Augusta, GA 30912, USA; 4 Department of General Surgery, Wyckoff Heights Medical Center, Brooklyn, NY 11237, USA; 5 The Hansjörg Wyss Department of Plastic Surgery, NYU Langone Health, New York, NY 10016, USA; 6 Department of Plastic & Reconstructive Surgery, University of California, Irvine, CA 92697, USA

**Keywords:** cleft lip, premaxillary setback, global surgery, craniofacial surgery

## Abstract

Various patients with complete bilateral cleft lip and palate present with a protruded premaxilla. Several techniques have been described for correctional repair of the projection with a plethora of unsatisfactory outcomes. This poses a challenge not only for the cleft team providing care but also for the patients and their respective families. Multiple patients suffer from residual deformities after inadequate primary repair, which increase surgical, financial, and psychological burden. Premaxillary setback with posterior vomerine ostectomy and complete bilateral cleft lip repair can promote alignment of the premaxilla with the maxillary prominences. To effectively address this challenging deformity, we describe a single-stage surgical technique that includes vomerine ostectomy posterior to the vomero–premaxillary suture, bilateral gingivoperiosteoplasties with complete bilateral cleft lip repair, and primary cleft rhinoplasty. Careful surgical planning is essential for adequate matching between the length of the protruded premaxilla and the extent of ostectomy. The described technique offers several advantages for the management of complete bilateral cleft lip with a projected premaxilla. It can be applied anywhere around the world and is most beneficial in underprivileged areas where patients suffer from restricted access to healthcare, absence of presurgical orthodontics and lack of sufficient resources.

## 1. Introduction

Clefts of the lip and/or palate occur in approximately 1 in 700 live births, with varying prevalence across regions. Early surgical intervention significantly enhances aesthetic and functional outcomes, psychosocial well-being, and quality of life while reducing the associated economic burden. However, limited healthcare access in underserved settings often results in late presentations, surpassing the recommended age for cleft lip and palate repair [1,2].

Patients in developing countries often lack access to an interdisciplinary cleft care team that allows for holistic care of the patient. A protruded premaxilla, even in developed countries, imposes a substantial challenge for cleft care providers. It regularly requires presurgical orthopedics, which in developing countries is often inaccessible to the patient, either technically or financially. Presurgical orthopedics often require long-term follow-up for proper outcomes which might be cumbersome due to the socioeconomic status of many of the caring families. For proper orthopedic care, patients require regular follow-up, occasionally twice per week, which is not easily achievable in many developing countries due to the scarcity of available transportation, security, and many more issues. Moreover, patients with a projected premaxilla that present at later stages in their life might have a completely ossified vomer bone. This makes the entire intervention of presurgical orthopedics ineffective [3].

To date, several techniques have been described to address wide complete bilateral cleft lip and palate (BCLP), either as staged procedures or with repair under tension. It is vital for surgeons performing these surgeries to be well equipped with the necessary knowledge to allow for constructive functional and aesthetic outcomes. Lip adhesion procedures have failed to address the deficient orbicularis oris muscle, resulting in a nonfunctioning lip and an uncorrected projected premaxilla. Moreover, it carries a higher risk of dehiscence and potential flap necrosis due to tight closure [3]. Excision of the premaxilla has also been seen to invariably lead to uncorrectable disfigurement, including the loss of the four central teeth [3]. A prominent premaxilla is a prevalent feature among patients with BCLP. Bilateral cleft lip (BCL) repair that does not address a severely projected premaxilla can result in weak muscle apposition and distorted prolabium with a widened inter-alar distance and stretched labial scars [4].

Vomerine ostectomy allows repositioning of the protruded premaxilla and decreases tension during and after cheiloplasty, resulting in reduced risk of wound dehiscence and infection [5]. However, premaxillary setback with vomerine ostectomy anterior to the vomero–premaxillary suture (VPS) has been associated with an increased risk of premaxillary necrosis, especially with concomitant nasolabial repair and alveolar gingivoperiosteoplasties (GPPs) [5]. This necrosis mainly stems from the fact that the major blood supply to the premaxilla arises from the dorsal nasal artery [3].

The aim of this article is to describe the first author (U.H.)’s approach to premaxillary setback that combines four procedures into one. This approach combines BCL repair with an ostectomy posterior to the VPS, bilateral GPPs, and primary cleft lip (CL) rhinoplasty. This single-stage procedure is shown in this manuscript with video footage and text description. This procedure can be safely performed on patients with a severely protruded premaxilla that have no access to interdisciplinary cleft care or to children with an ossified vomer bone that have no choice otherwise.

## 2. Materials and Methods

### 2.1. Surgical Technique

It is imperative to always keep in mind the following tenets for desirable aesthetic and functional outcomes when dealing with a patient presenting with a protruded premaxilla. The anteriorly projected premaxilla should be displaced posteriorly, ensuring its proper alignment with the secondary palatine shelves. The Cupid’s bow should be aligned and symmetrical such as both peaks are equidistant from the nadir. The peaks of the Cupid’s bow should be symmetrical to the respective nostril bases. The orbicularis oris muscle should be properly restored, ensuring its continuity throughout the upper lip, avoiding residual deficiency or notching. The surgeon should secure the symmetry of the philtral ridges with adequate fullness of the philtral tubercles. Finally, it is imperative to support and project the top of the nose, ensuring symmetrical outflaring of the medial crural footplates, with symmetrical width and shape of the nostrils.

Achieving a setback of the premaxilla is crucial in preventing the development of anterior vectors during the healing process, which could otherwise lead to issues like lip dehiscence and widened scarring. Bilateral GPP plays a key role in ensuring stability and the correct alignment of the premaxilla with the secondary palatine shelves. This alignment is essential for the success of subsequent cleft palate repair procedures. Insufficient retropositioning of the premaxilla may lead to a failed cleft palate repair, often resulting in the formation of anterior fistulas.

While the ideal timing for the repair of this procedure is a subject of debate, studies have demonstrated its safety and effectiveness in patients as young as 4 months old [3]. Moreover, its efficacy extends to older patients, reaching up to 10 years of age, even in cases involving ossified vomer. This is particularly noteworthy as alternative techniques like lip adhesion might have proven ineffective in such scenarios.

Surgical planning is centered on the physical examination. Patients who benefit most from a premaxillary setback are those with a premaxillary protrusion that is more than 10 mm in a setting where other treatment options are not available. This includes patients with a premaxilla that is unamenable to presurgical orthopedics, patients with no access to presurgical orthopedics, and older patients presenting with an ossified vomer bone that prevents adequate lip closure. Patients with contracted secondary palatine shelves may require manual expansion prior to premaxillary setback. The length of the ostectomy correlates to the extent of the protrusion of the premaxilla.

The described surgery is performed under general anesthesia with a preformed micro-cuffed oral endotracheal tube (ETT) to minimize any interference with the surgical site access. The ETT is affixed to the chin using fluid-resistant tape, precisely positioned at the midline just beneath the lower lip. This meticulous securing method is employed to maintain the natural shape and position of the resting lower lip. The patient is positioned in a supine position, with the neck extended using a shoulder roll, and the head comfortably supported by a soft silicone gel headrest. To mitigate the risk of airway fires, a moist throat pack secured with a silk suture is carefully placed around the ETT. Intravenous administration of prophylactic antibiotics is given for added precaution.

### 2.2. Surgical Marking

**Figure 1:**
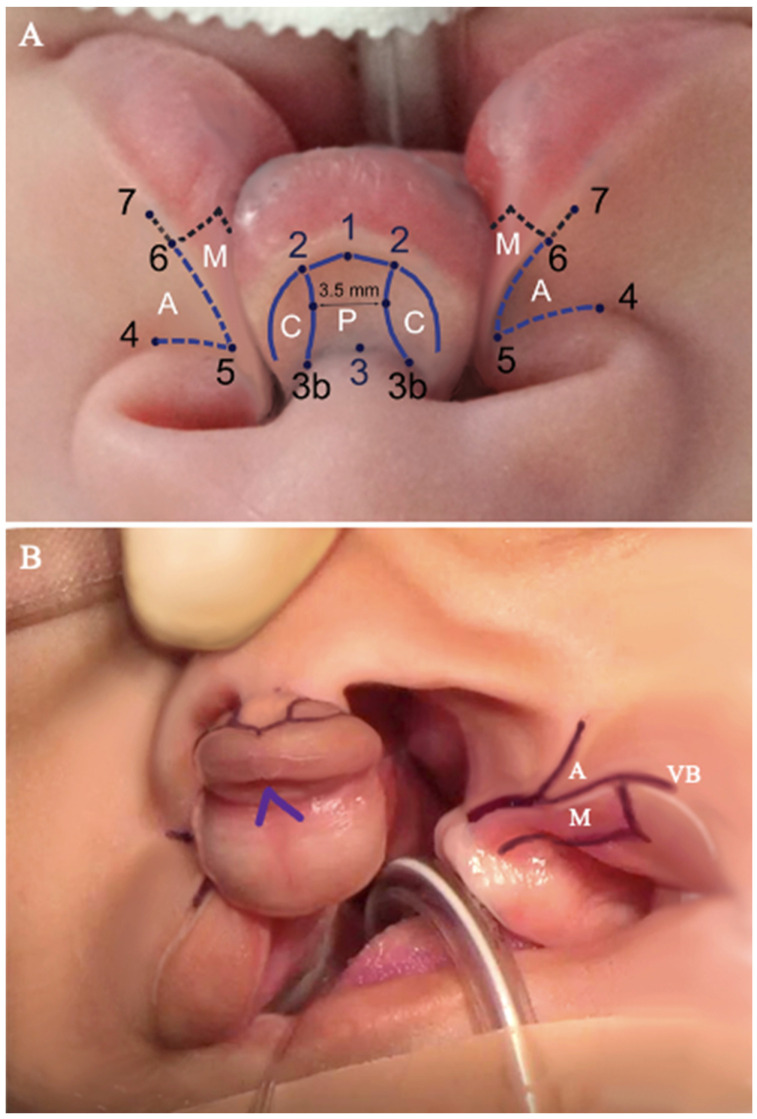
(**A**). Surgical markings performed with a fine-tip surgical marking pen, as described; (**B**). advancement (A) flap, mucosal (M) flap, and vermilion border (VB) flap are marked as shown, along with a 1.5 mm inverted V-shape buccal mucosal flap is marked in the midline to re-create the frenulum.

Surgical markings for a comprehensive BCL repair are meticulously performed using a fine marking pen, as detailed in Figure 1 [3] (Supplementary Video S1):

1. As illustrated in Figure 1A, the lowest point of Cupid’s bow, which is more clearly observed from a skyline perspective, is carefully indicated just above the vermilion white line in the prolabial flap (1). Utilizing a caliper, the two elevated points of Cupid’s bow (2) are precisely marked at 2 mm from the lowest point. Subsequently, these marked points are interconnected by a dashed line.
2. The angle formed by the columellar and labial regions is identified by marking it precisely at the midpoint of the crease (3).
3. In cases where the premaxilla undergoes significant rotation, causing distortion in the accurate markings of the nadir of Cupid’s bow, an alternative identification method involves drawing a straight perpendicular line from the mid-columellar point to the low point (1).
4. The delineation of philtral ridges involves connecting the outflaring of the footplates (3b) to the peaks of Cupid’s bow (2) through a curvilinear concave segment. This connection is made with precision, ensuring that the narrowest distance at the vertical midline of the philtral column measures 3.5 mm. Achieving a clear view of the outflaring footplates is facilitated by gently retracting the nostrils using a double-prong retractor. The resultant structure forms the prolabial (P) flap, alongside which lie the columellar (C) flaps. These columellar flaps will undergo trimming and rotation to faithfully recreate the outflaring of the medial crural footplates. It is important to note that a significant portion of the dry vermilion in the prolabial area will be discarded in this process.
5. Both the alar bases (4) and the most medial points (5) are meticulously marked on both sides.
6. The most medial edge of the three-dimensional structure of the vermilion border is marked on each side of the lip, representing the peak of Cupid’s bow (6).
7. Advancement (A) flaps are meticulously outlined by connecting points (4), (5), and (6) on both sides. It is crucial to note that these (A) flaps are intentionally limited, ensuring they do not extend beyond the nostril sill creases to prevent any undesirable scarring in the alar crease (Figure 1B).
8. Mucosal (M) flaps are meticulously delineated on both sides by drawing a parallel line to the white skin incision at the level of the wet–dry junction, extending into the gingivobuccal sulcus. In cases involving premaxillary setback, these mucosal flaps are discarded. However, in non-premaxillary setback bilateral cleft lip repair, they play a crucial role in recreating the nasal floor, as illustrated in Figure 1B.
9. Vermilion border (VB) flaps are carefully marked on both sides, ensuring that the edges (7) are positioned 2.5 to 3 mm from the peaks of Cupid’s bow (6) as depicted in Figure 1B. The intentional inclusion of an additional 0.5 to 1 mm in the flap design facilitates sufficient rotation of VB flaps, contributing to the creation of a natural curvature that enhances the contour of Cupid’s bow.
10. A 1.5 mm inverted V-shaped buccal mucosal flap is meticulously marked at the midline to recreate the frenulum, as illustrated in Figure 1B.
11. To ensure optimal visualization of the vomer bone, a Dingman retractor is used.
12. The VPS is precisely outlined. For the posterior vomerine ostectomy, a “conjoined double Y” marking is strategically placed, with the anterior part positioned at least 5 mm posterior to the VPS. This strategic placement minimizes interference with the premaxillary blood supply, reducing the risk of premaxillary necrosis commonly observed in cases with premaxillary setbacks involving anterior vomerine ostectomies. The marking is extended with a Y-shape on both ends, ensuring a tension-free dissection during the ostectomy and providing ample exposure of the vomer bone. Preserving the integrity of the vomerine flaps is vital, as it can impact closure and midfacial growth.

### 2.3. Local Anesthesia

Infraorbital and external nasal nerve blocks are meticulously administered. The local anesthetic mixture comprises equal volumes (1:1) of bupivacaine 0.25% and lidocaine 0.5%, combined with 1:150,000 epinephrine. The maximum allowable dose is carefully determined based on the patient’s weight, with a guideline of 1 cc/kg for children under 12 years of age. This calculation is performed by the anesthesiologist and verified by the surgeon.

The mixture is injected bilaterally around the infraorbital foramina and the external nasal nerves. Additionally, it is introduced under the vomerine mucoperiosteal flaps, at the incision site for the GPPs, along the medial edge of the secondary palatine shelves, into the prolabial flap, at the alar base, and on the surgical sites of the upper lip. To optimize the vasoconstrictive effects of epinephrine and minimize postoperative discomfort, the incision is delayed until 12 min after the injection. (Supplementary Video S1).

### 2.4. Posterior Vomerine Ostectomy

The scope of the ostectomy is predetermined preoperatively and corresponds to the anterior projection of the premaxilla, with the goal of aligning it with the alveolar ridges to establish continuity in the maxillary arch (Supplementary Video S2).

**Figure 2:**
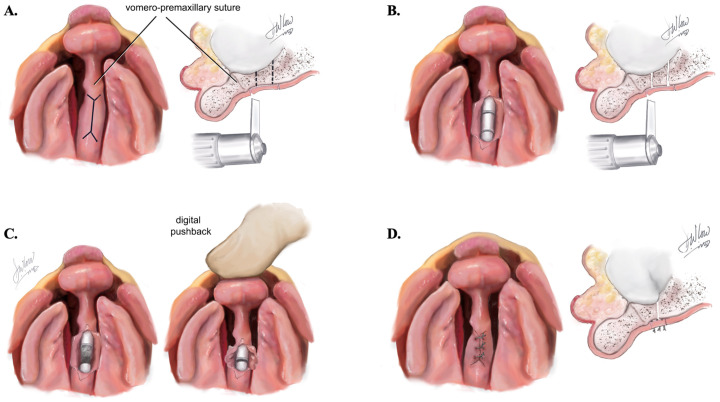
(**A**)**.** “Conjoined double Y” marking with the anterior margin being at 5 mm posterior to the center of the VPS. (**B**). Ostectomy is performed with horizontal cuts on the anterior and posterior portion of the vomer segment to be removed. (**C**). Digital pushback by applying pressure to the premaxilla and mobilizing it posteriorly. Critical for the premaxilla to align with the secondary palatine shelves. (**D**). Suturing of the vomerine mucoperiosteal flaps.

After the VPS is identified, a “conjoined double-Y” incision is meticulously created using a 15-blade or Colorado^®^ microdissection needle. This incision involves a vertical cut along the caudal crest of the vomer bone, extending in a Y-shape on both ends. The anterior margin of the incision is positioned at least 5 mm behind the center of the VPS to preserve the premaxillary blood supply (Figure 2A).

Subsequent steps involve incising the mucosa and raising bilateral flaps laterally from the vomer and septum in a subperiosteal plane. Precise dissection of the flaps is crucial to maintain sufficient blood supply to the mucosa and the vomer, as well as to prevent damage to the nasal septal cartilage and the midface growth center [5].

Once the vomer bone is adequately exposed, the ostectomy is conducted using a 4 mm or 6 mm osteotome or an oscillating saw. Horizontal cuts are made on the anterior and posterior portions of the segment to be removed. In cases where the premaxilla projection is angulated or laterally deviated, the ostectomy is directed at an angle that effectively realigns the premaxilla with the maxillary arch (Figure 2B).

The cut section of the vomer bone is gently removed using bone rongeur or pituitary forceps, with careful attention to avoiding damage to superior anatomical structures. Twisting motions are consciously avoided to prevent any harm to the skull base [3,4,5].

Following the ostectomy, pressure is applied to the premaxilla to ensure sufficient laxity for posterior mobilization, aligning it with the secondary palatine shelves (Figure 2C). Meticulous suturing of the vomerine mucoperiosteal flaps follows, sutured with 5-0 Vicryl© on an RB-1 needle (Figure 2D).

### 2.5. Bilateral Gingivoperiosteoplasties

**Figure 3:**
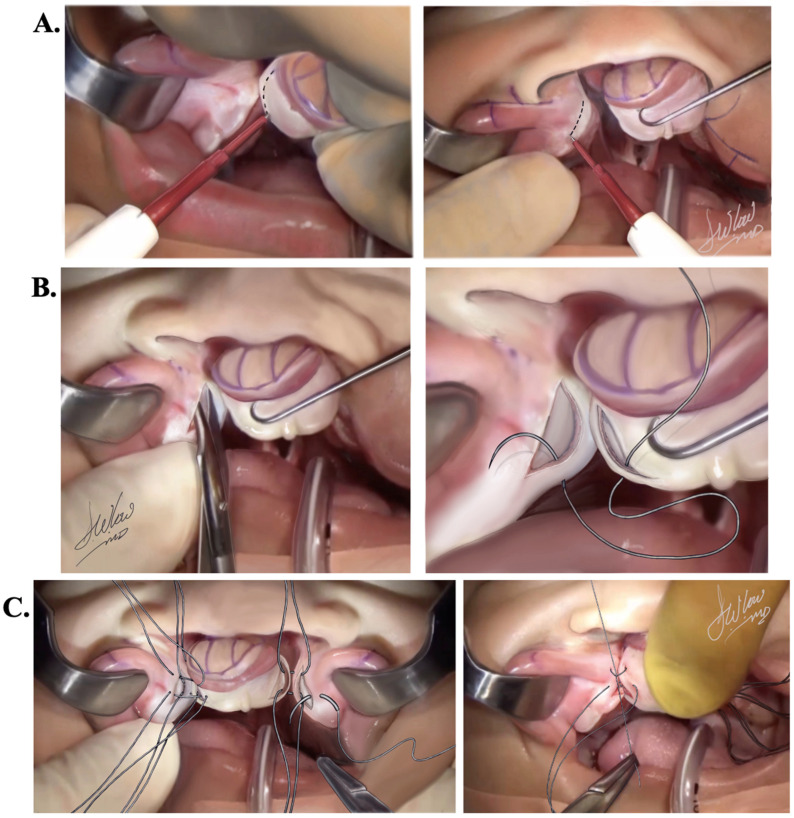
(**A**). A Colorado^®^ microdissection needle is used in cutting mode to make mucoperiosteal incisions as shown along the dashed lines. (**B**). Mucoperiosteal flaps are elevated and sutured with 4-0 Vicryl^®^ on an RB-1 needle following an “*8-point fixation system*”. (**C**). Four anchor sutures are placed at the level of the palatine shelves, two on each side of the GPP. Once tied, four anchor sutures are placed at the level of the buccal mucosa, two on each side of the GPP, and tied.

Bilateral gingivoperiosteoplasties (GPP) are undertaken as a preliminary step preceding cleft lip repair, with the dual objectives of stabilizing the premaxilla and mitigating anterior vectors of stress on the repaired lip. Employing a Colorado^®^ microdissection needle in cutting mode, set at 5 W, mucoperiosteal incisions are meticulously made, ensuring precision to cut only through the periosteum and not the bone (Figure 3A). Subsequently, the flaps are carefully elevated in a subperiosteal plane using a Freer instrument to expose the underlying bone.

During this process, the surgical assistant applies digital pressure on the premaxilla, maintaining parallel alignment with its horizontal plane until it aligns seamlessly with the secondary palatine shelves. Caution is exercised to prevent any rotation of the premaxilla that might result in postoperative under- or over-rotation (Figure 3B).

Suturing of the mucosa and periosteum follows, utilizing 4-0 Vicryl^®^ (Ethicon, Cincinnati, OH, USA) on an RB-1 needle, employing an “8-point fixation system”. Four anchor sutures are strategically placed on each side of the GPP—two on the palatal side and two on the buccal side (Supplementary Video S2). Continuous pressure is applied throughout the suturing process to prevent premaxillary movement, which could lead to a gap between it and the lateral palatal elements. The pressure is maintained until all anchor sutures are tied, ensuring the stability of the premaxilla (Figure 3C).

In cases of complete BCL with a projected premaxilla and an intact, unrepaired palate, access to the vomer is facilitated through the anterior margin of the palate, typically covered by a membranous lining that overshadows the vomer. For patients with a previously repaired palate, anterior mucoperiosteal flaps are meticulously elevated from the level of the first molar on one side to the level of the first molar on the contralateral side. This allows access to the vomer bone, and these flaps are carefully sutured at the conclusion of the vomerine ostectomy procedure to ensure a thorough palate repair and prevent potential fistula formation [3].

### 2.6. Lip Dissection

**Figure 4:**
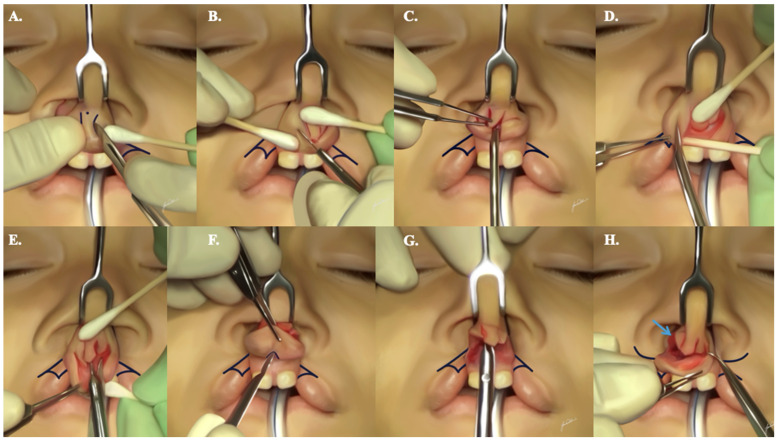
A step-by-step demonstration illustrating the appropriate sequence of incisions to allow for proper lip dissection, as described. Incision starts in subfigures (**A**,**B**) along the marked lines followed by subsequent dissection in subfigures (**C**–**E**). Subfigures (**F**,**G**) show the inferior incision and dissection of the labial frenulum to recreate the philtral dimple. Subfigure (**H**) shows the relaxing incision (blue arrow) that is crucial for mobilization of the flaps.

**Figure 5:**
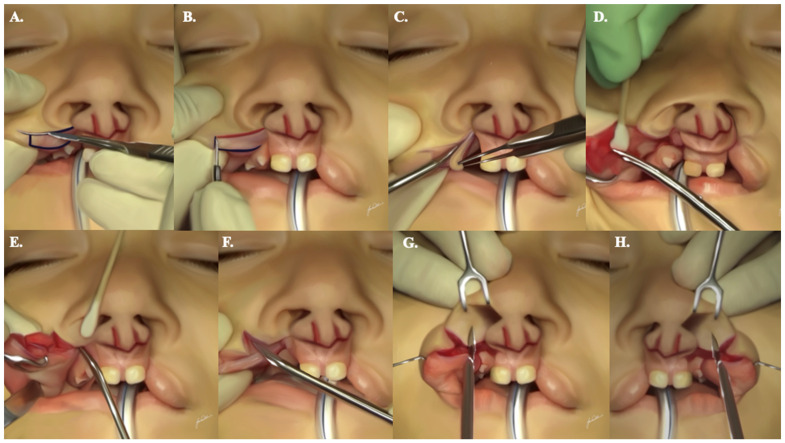
A step-by-step demonstration illustrating the appropriate sequence of incisions to allow for proper lip dissection, as described. Initial incisions are done along the lines shown in subfigures (**A**,**B**), followed by subsequent dissection of the lip according to the above-described technique in subfigures (**C**–**H**).

Paying attention to the markings made as in Figure 1, the elevation of the prolabial (P) and columellar (C) flaps, followed by the advancement (A) and vermilion border (VB) flaps, involves a systematic approach (Supplementary Video S3) [3]:

1. Raise the (P) flap and adjacent (C) flaps in a subdermal plane up to the level of the columellar crease using a #15 blade. Ensure that the thickness of the flap includes both skin and subcutaneous tissue. The (C) flaps should be adequately released to allow a 90-degree lateral rotation with minimal force (Figure 4A–C).
2. Trim the premaxillary mucosa, discarding most of the dry vermilion (Figure 4D). Preserve a vestigial 1.5 mm inverted-V mucosal triangle to reconstruct the frenulum. Thinning of this premaxillary mucosa is crucial to avoid undesirable excess tissue in the area.
3. Slightly thin the central part of the (P) flap subcutaneously to recreate the philtral dimple. Ensure the preservation of the subdermal blood supply to the flap (Figure 4E–H).
4. Raise the (A) and (VB) flaps as marked using a #15 blade. Ensure sufficient flap thickness to allow for the application of transverse and subcutaneous sutures (Figure 5A,B).
5. Elevate and discard the (M) flap (Figure 5C).
6. Make a 2 to 3 cm supra-periosteal gingivobuccal sulcus incision bilaterally to facilitate the medial mobilization of (A) flaps.
7. Carefully dissect the orbicularis oris muscle from the overlying skin, starting at the level of the alar base and moving in a downward and curvilinear direction toward the vermilion border. Ensure that the muscle dissection does not extend laterally beyond 10 mm from the cleft and beyond the vermilion border, preserving the anatomical and functional integrity of the lip (Figure 5D–H).
8. The arrow shown in Figure 5H shows the relaxing incision in the lateral aspect of the C flap that extends into the membranous septum. This incision, when performed bilaterally, will allow for the inferior mobilization of the prolabial flap to facilitate the execution of the step-down technique without the undesirable effect of blunting of the infra-tip lobule or superior displacement of the newly created Cupid’s bow.

### 2.7. Primary Rhinoplasty

The procedure for cartilage-sparing CL rhinoplasty involves columellar access, delicately separating the nasal skin from the underlying lower lateral cartilages to facilitate their medial repositioning. Employing sharp ribbon-handle scissors, dissection is performed between the cartilage and the overlying skin from the columella to the nasal dome. It is essential for the surgeon to exercise caution and refrain from wide-spreading maneuvers that could potentially damage the delicate lower lateral cartilages. The dissection is halted at the level of alar creases to safeguard the structural integrity of these cartilages.

For the alar base flaps, the alar rim is gently retracted using a wide double hook. An incision is made at the upper edge of the vibrissae (nasal hairs), approximately 2 mm posterior to the posterior border of the lower lateral cartilage, running parallel to the alar rim. This technique is employed to preserve the integrity of the lower lateral cartilages. It is crucial to avoid accessing cartilage dissection through a lateral approach, specifically via the alar base, as this poses a risk of disrupting the natural contour of the ala and causing buckling of the alar rim.

### 2.8. Sliding V-Cheiloplasty

**Figure 6:**
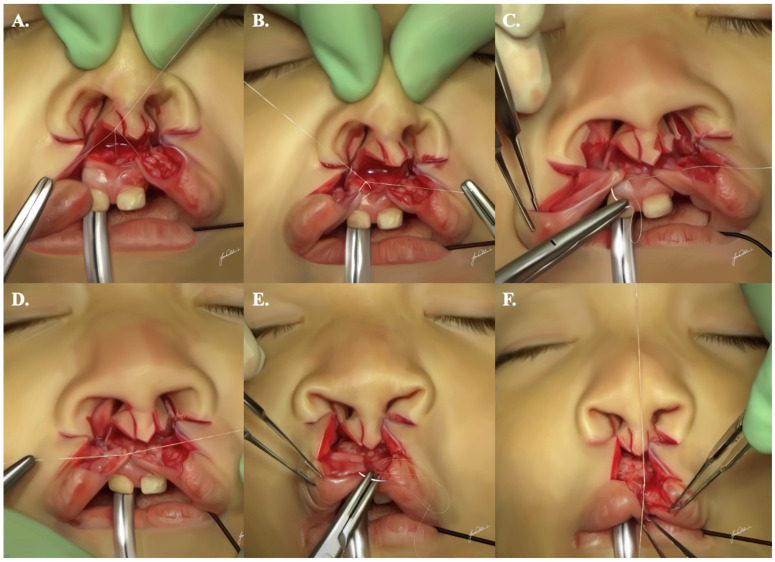
A step-by-step demonstration illustrating the appropriate suturing sequence needed to allow for proper cheiloplasty, as described. Subfigures (**A**,**B**) show the triangular suture necessary for the proper reconstruction and recreation of the frenulum. Subfigures (**C**,**D**) show the proper approximation of the created flaps. Subfigures (**E**,**F**) demonstrates the repair of the orbicularis oris muscle.

Buccal mucosal flaps are meticulously sutured to the premaxilla using interrupted 5-0 Vicryl^®^ on an RB-1 needle. The recreation of the frenulum involves a triangular suture applied between the upper and medial edges of the buccal flaps and the vestigial inverted mucosal flap preserved on the premaxilla (Figure 6A–D).

The sliding V-cheiloplasty technique is then meticulously executed to repair the orbicularis oris muscle, utilizing 5-0 Vicryl^®^ on RB-1. Applying at least five to six anchor sutures in a V-shaped fashion, each positioned 2 mm inferior and medial to the preceding suture, enables lip lengthening and medial mobilization of the alae. This approach ensures a symmetrical and precise repair of the orbicularis oris muscle, contributing to the creation of the philtral tubercle (Figure 6E,F).

### 2.9. Primary Rhinoplasty (Continued)

**Figure 7:**
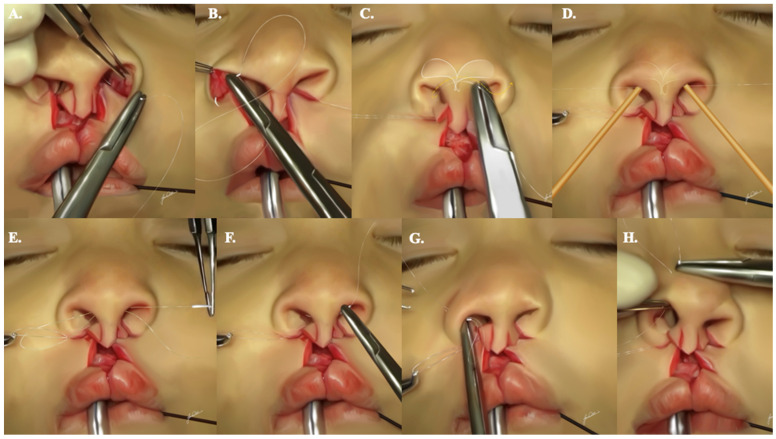
A step-by-step demonstration illustrating the appropriate suturing sequence needed to allow for proper primary rhinoplasty, as described. Subfigures (**A**,**B**) show the alar base flap suspending sutures. Subfigures (**C**,**D**) show the two interdomal sutures. Subfigures (**E**,**F**) show the alar crease transfixion sutures. Subfigures (**G**,**H**) show suspending sutures behind the rim of the soft triangle.

An alar base flap suspending suture, utilizing 4-0 Vicryl^®^ on an RB-1 needle, is subsequently applied to both alar lobule bases. It is tied in a loop fashion to bring them into approximation (Figure 7A,B). However, this suture is not fully tightened until the completion of the primary cleft lip rhinoplasty, ensuring bilateral nostril symmetry. This strategic approach provides ample support for the medialized alar lobules and counteracts the lateralizing vectors of the healing process.

A sequence of crucial sutures is employed to guarantee a comprehensive cleft lip rhinoplasty with optimal tip support, tip projection, and nostril symmetry (Supplementary Video S4):

1. Two interdomal sutures are delicately positioned on each side, addressing the upper and lower edges of the nasal dome with 4-0 Monocryl^®^ on a PS-2 needle. The initial suture is strategically placed at the junction of the domes and the medial crura of the lower lateral cartilages. Subsequently, the second suture is elevated superior to the first, contributing to the creation of a more refined nasal tip. This meticulous approach ensures not only satisfactory anterior nasal tip projection but also an enhancement of tip support (Figure 7C,D).
2. To enhance alar symmetry and eliminate potential dead space resulting from alar base flap dissection, two to three alar crease transfixion sutures are applied on each side, utilizing 6-0 Monocryl^®^ on a P-1 needle (Figure 7E,F).
3. Addressing the redundancy in the soft triangle, one to two suspending sutures (4-0 Monocryl^®^ on a PS-2 needle or straight Keith needle) are strategically placed 2 to 3 mm behind the rim of the soft triangle. These sutures exit through the upper lateral cartilage on either the ipsilateral or contralateral sides. Following this, the suture is reentered through the same exit hole and directed toward the lower lateral cartilage, exiting along its lower rim. It is crucial to tie these sutures without causing alar rim notching (Figure 7G,H).
4. A stabilizing transfixion suture at the level of the transverse crura is implemented using 4-0 Monocryl^®^ on a PS-2 needle.
5. For anchoring the tip of the (A) flaps to the columellar bases bilaterally, transverse sutures are meticulously applied. Using 5-0 Vicryl^®^ suture on an RB-1 needle, these sutures are positioned 2 mm behind the tip of the advancement flap. This ensures a secure and balanced outcome.

### 2.10. Cheiloplasty (Continued)

Ensuring optimal outcomes necessitates attention to Cupid’s bow symmetry and alignment, philtral length, and the aesthetic three-dimensional (3-D) shape. A prolabial flap of insufficient length can lead to the superior displacement of the newly formed Cupid’s bow, potentially resulting in an unappealing outcome due to the height disparity between the short prolabial flap and the reconstructed Cupid’s bow. Additionally, a short flap may cause blunting of the nasal tip and a shortened or nonexistent columella.

**Figure 8:**
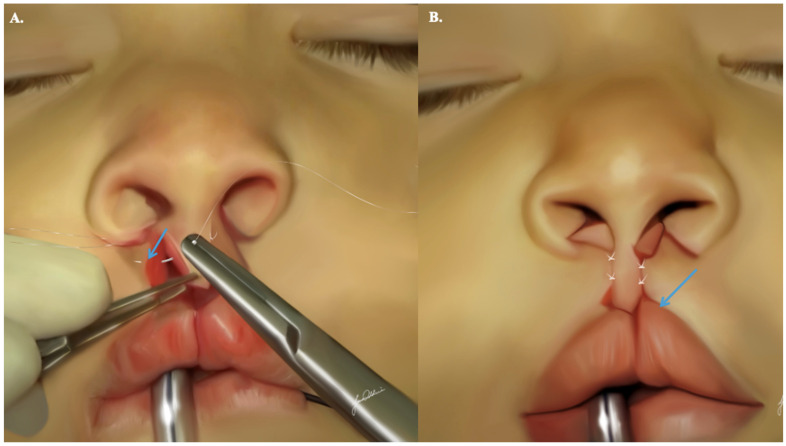
Stepdown technique: allows lengthening of the available skin in the prolabial flap. This facilitates correction of symmetry between the height of the prolabial flap and Cupid’s bow. Subfigure (**A**) shows the exact position of needle insertion (blue arrow) to allow for proper symmetry of the prolabial flap and Cupid’s bow. Subfigure (**B**) shows the final resu lt if the stepdown technique is executed properly with the blue arrow portraying this symmetry.

To address these concerns, the flap can be lengthened by incorporating relaxing incisions extending from the lateral side of the (C) flaps into the membranous septum. This allows for the inferior mobilization of the flap toward the newly created Cupid’s bow. The stepdown technique further compensates for the height difference between the prolabial flap and the newly established Cupid’s bow by extending the available skin in the flap (Supplementary Video S3). The initial suture on the prolabial flap is strategically placed 1 mm below the columellar–labial crease, angled 2 to 3 mm inferiorly toward the advancement flap. The second suture, situated 2 mm below the columellar–labial crease, is also angled 2 to 3 mm inferiorly. At this stage, the remaining non-sutured portion of the prolabial flap aligns well with the upper edge of the vermilion border, ensuring tension-free approximation during the reconstruction of Cupid’s bow (Figure 8).

For the alignment, symmetry, and continuity of Cupid’s bow and philtrum, triangular sutures are applied between the lateral peaks of Cupid’s bow (points 6 on Figure 1A) and the prolabial low point (point 1 on Figure 1A) using 6-0 Monocryl^®^ on a P-1 needle. Bilateral anchor sutures are then employed to rotate the lateral margins of VB flaps (points 7 on Figure 1A) and match them to the prolabial vermilion high points (points 2 on Figure 1A). The extended VB flaps allow sufficient rotation to provide a natural curvature to the contour of Cupid’s bow. These meticulous steps are crucial for ensuring the alignment, symmetry, and continuity of Cupid’s bow.

### 2.11. Primary Rhinoplasty (Continued)

The (C) flaps undergo trimming to recreate the nostril sill and replicate the outflaring observed in natural footplates. The quadrangular suture, a single application using 6-0 Monocryl^®^ on a P-1 needle, is employed to ensure symmetric and tension-free closure of the nostril sill. This suture is executed in the following sequence:

1. The needle penetrates the skin, exiting subcutaneously at the upper edge of the (A) flap.
2. It then traverses, entering and exiting subcutaneously at the medial edge of the alar base flap.
3. Subsequently, it is inserted and exits subcutaneously at the opposing edge of the (C) flap.
4. Finally, the needle is inserted subcutaneously on the upper edge of the advancement flap and exits through the skin. This technique facilitates the convergence of the three anatomic structures with a single suture, ensuring a seamless closure.

### 2.12. Final Remarks

For skin closure, cutaneous or intradermal sutures are employed, utilizing 6-0 Monocryl^®^ or PDS^®^ sutures. If required, the epidermis layer is closed with 5-0 or 6-0 Fast Absorbing Plain Gut^®^ sutures on a PC-1 needle. The utilization of absorbable sutures eliminates the necessity for a return visit to the operating room for suture removal, proving particularly advantageous in low- and middle-income countries.

## 3. Results

Our proposed intervention aims for a safe and tension-free repair of a complete BCL and nose, with adequate muscle approximation, to enhance both functional and aesthetic outcomes [3]. The posterior vomerine ostectomy allows to maintain and preserve adequate blood supply to the area for optimal vomer bone repopulation and subsequently normal facial growth.

Nine important desirable outcomes for repair are needed: posterior displacement of an anteriorly projected premaxilla, proper alignment of the premaxilla with the secondary palatine shelves, proper alignment of Cupid’s bow, restoration of the orbicularis oris muscle, symmetry of the philtral ridges, adequate fullness of the philtral tubercle, adequate projection and support of the nasal tip, symmetrical outflaring of the medial crural footplates, and symmetrical width and shape of the nostrils [3]. These nine tenets may not be all achievable and outcomes should be tailored accordingly on a case-by-case basis.

**Figure 9:**
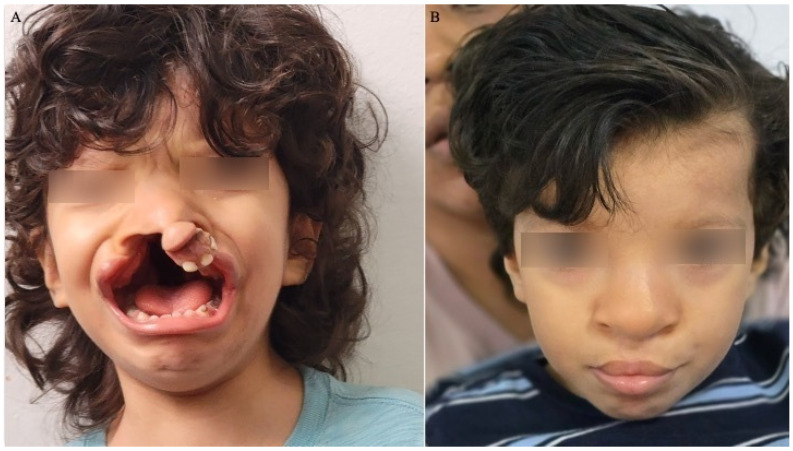
(**A**). Pre-surgical picture of a patient with a significantly misaligned and protruded premaxilla. (**B**). Postoperative picture of the same patient after a single-stage premaxillary setback was performed.

Figure 9 illustrates the case of a three-year-old boy who came to our attention with a severely misaligned and protruding premaxilla (Figure 9A). Notably, his vomer bone had undergone complete ossification, rendering him unsuitable for conventional interventions such as NAM or orthognathic/orthodontic procedures. Prior to seeking our assistance, he had remained secluded from society without any prior intervention. However, in September 2023, he underwent the technique we described, and we subsequently conducted a follow-up examination after six months in March 2024 (Figure 9B).

## 4. Discussion

Premaxillary protrusion has always caused a burden on both the treating physician and the treated patient. It complicates the management and repair of BCLP and if not approached properly it can lead to devastating consequences. Several techniques have been proposed to properly deal with premaxillary protrusion, from presurgical orthopedics to surgical repair. However, functional, anatomical, and aesthetic outcomes should always be taken into consideration. Surgeons should aim to preserve functional and anatomical integrity to allow for midfacial growth and orthognathic maturation [3,6].

In outreach settings, where access to presurgical orthopedics is costly and scarce, it is vital to provide patients with a safe and effective intervention that will allow them to reintegrate into their society seamlessly. In our outreach medical programs, the average age at time of repair for patients with BCLP and protruded premaxilla is 13 months. This age greatly depends on time of presentation and on the need and availability of presurgical interventions [7].

The management of a protruded premaxilla should be evaluated on a case-by-case basis. It is vital to assess access to the vomer bone in all patients presenting with a protruded premaxilla. Access to the vomer bone can either be clear with a full view of the bony/cartilaginous area, severely restricted and inaccessible, or clear view but with a skewed vomer bone. Patients presenting with clear access to the vomer bone will benefit greatly from our proposed intervention and will potentially have an eventless surgery. Patients presenting with an inaccessible vomer bone due to the collapse of the secondary palatine shelves will require expansion for vomer access. Presurgical orthodontics and Naso-Alveolar Molding (NAM) can be used to attempt expansion of the collapsed secondary palatine shelves. Custom made patient-specific intraoral maxillary expanders may expand the palatine shelves and reduce premaxillary protrusion. However, in case presurgical orthodontics fail to expand the palatine shelves, manual expansion with a 4 × 4 prong retractor may be used to gain access to the vomer bone [3]. Patients presenting with a skewed vomer bone should always be evaluated meticulously due to the need for an angled ostectomy. It is important for the vomerine ostectomy to be commensurate with the anterior projection of the premaxilla, aiming to align the premaxilla with the alveolar ridges to achieve continuity of the premaxillary arch [3].

Like many surgical procedures, our proposed intervention carries both advantages and disadvantages. The benefits are manifold, primarily centered on its capacity to achieve both aesthetic and functional objectives, thereby ensuring comprehensive enhancement. These desirable outcomes include realignment of the premaxilla, effectively positioning it posteriorly to harmonize with the secondary palatine shelves; achieving proper alignment and symmetry of the Cupid’s bow, ensuring equidistant peaks for aesthetic equilibrium; establishing symmetry between the peaks of the Cupid’s bow and the corresponding nostril bases; restoring continuity of the orbicularis oris muscle across the upper lip to prevent residual deficiency or notching; ensuring symmetry of the philtral ridges and adequate fullness of the philtral tubercles for a natural appearance; providing ample support and projection to the nasal tip, achieving symmetrical outflaring of the medial crural footplates, and maintaining uniform width and shape of the nostrils; and effectively repositioning the premaxilla to seamlessly align with the secondary palatine shelves, thereby optimizing functional alignment [3].

Conversely, the disadvantages stem from the improper execution of the described surgical techniques. Inadequate stabilization of the premaxilla may occur if it is not properly secured to the lateral secondary palatine shelves during bilateral GPP. There is a theoretical risk of premaxillary necrosis due to potential damage to the blood supply, although instances of premaxillary ischemia have not been reported with premaxillary setback with vomerine ostectomy at least 5 mm posterior to the VPS, unlike with anterior premaxillary setback and anterior vomerine ostectomy. Lip dehiscence may result if the healing vectors are not appropriately managed, but this risk can be mitigated by employing layered lip closure and ensuring sufficient premaxillary setback. Over-rotation of the premaxilla is also possible, particularly if proper technique is not observed during GPP. Concerns about inadequate long-term craniofacial growth exist; however, current research lacks definitive evidence demonstrating the technique’s impact on facial growth. There is a risk of neurological damage with overzealous removal of vomerine bone, potentially affecting the cribriform plate, but careful visualization and gentle ostectomy techniques can help prevent such complications [3].

More importantly, postoperative care for the patient should revolve around all parameters that enhance recovery and prevent any inadvertent damage to the newly repaired area. Postoperatively, patients are initially placed on a liquid diet until discharge on postoperative day 1, contingent upon achieving adequate per oral intake. Subsequently, a pureed diet is recommended for the following 2 weeks post-discharge, after which a return to a regular diet is permissible. Antibiotic treatment involves a 5-day course of oral antibiotics with Staphylococcus aureus coverage. Additionally, topical antibiotic ointment is prescribed for severe crusting, applicable at the nasal sill using a Q-tip for one week.

To mitigate the risk of lip trauma, elbow immobilizers are routinely employed in children and can be removed under supervision. Lip care instructions include circular massage of the upper lip, specifically over the lip scar, commencing 6 weeks post-surgery. Ideally, these massages should be performed five to six times daily for a duration of 5 to 6 months to prevent hypertrophied scar development. Patients are advised to limit sun exposure and apply sunscreen to the scar for one year post-surgery to prevent hyperpigmentation. Follow-up appointments are scheduled at 7, 30, and 90 days postoperatively, followed by yearly appointments with both an experienced surgeon, a pediatrician, and an orthodontist in order to ensure proper healing and closure of the palatine shelves, plan the repair of the cleft palate at the appropriate time, and proper overall growth and development.

Much like any surgical technique, there are a set of complications that should be anticipated and cared for in case they materialize. In addition to the potential complications inherent in cleft lip repair, such as bleeding, wound infection, and hypertrophic scarring, unsatisfactory outcomes may arise, particularly when improper techniques are applied. Poor stabilization of the premaxilla may result from inadequate securing to the lateral secondary palatine shelves during bilateral GPP.

Theoretical risks of premaxillary necrosis exist due to potential damage to the blood supply, although our experience with premaxillary setback with vomerine ostectomy at least 5 mm posterior to the VPS has not yielded instances of premaxillary ischemia. Lip dehiscence is a conceivable complication if anterior vectors of healing are not considered; however, layered lip closure and sufficient premaxillary setback can mitigate this risk. Over rotation of the premaxilla is a technique-dependent concern, emphasizing the need for careful attention during GPP. Theoretical complications, such as injury to the cribriform plate and neurological damage, may arise from overzealous removal of vomerine bone; however, adequate visualization and gentle ostectomy can help surgeons avoid such issues.

Historically, some studies have suggested that primary premaxillary setback has been associated with midface retrusion causing long-term consequences, while other studies found that correctly retropositioning the premaxilla contributed to harmony with the mandible. Exploring the craniofacial growth patterns in these patients presents a significant opportunity as the prominent premaxilla contributes an additional factor to their growth profile. The authors are dedicated to recording the ongoing development patterns of their patients and are presently engaged in a research project that examines the facial growth of individuals who have undergone premaxillary setback.

We are currently comparing different facial growth parameters between patients who underwent premaxillary setback and previously reported Caucasian American non-cleft-standard patients. This study is also being standardized against age-matched controls of similar racial backgrounds. We have so far found no significant difference between our sample mean and the American sample mean across five different facial parameters for children aged 4–7 and 5–12. The study is still ongoing, and our results are being updated accordingly.

## 5. Conclusions

This technique allows for a single-stage premaxillary setback with posterior vomerine ostectomy, bilateral GPPs, and BCL repair with primary CL rhinoplasty. It provides many advantages, including the improvement of patients’ quality of life and social integration, especially in underserved settings where access to healthcare and resources are limited. It also offers a valuable alternative for patients with limited access to pre-surgical orthopedics and/or inconsistent follow-up, as well as for older patients presenting with an ossified vomer bone.

## Data Availability

The data presented in this study are available on request from the corresponding author. The data are not publicly available due to the presence of proprietary information and ongoing research work.

## Supplementary Materials

The following supporting information can be downloaded at: https://doi.org/10.5281/zenodo.10908747, Video S1. A video showing the surgical markings followed by the external nasal and infraorbital nerve blocks with local infiltration. Video S2. A video showing vomerine ostectomy posterior to the VPS with bilateral GPP. Video S3. A video showing bilateral CL repair. Video S4. A video demonstrating cartilage-sparing CL rhinoplasty technique.
